# Solving strategic problems of medicine of the future on the basis of the foresight methodology

**DOI:** 10.1186/1878-5085-5-S1-A65

**Published:** 2014-02-11

**Authors:** Natalia D Pankratova, Rostyslav V Bubnov

**Affiliations:** 1 Institute for Applied Systems Analysis NTU "KPI", Kyiv, Ukraine; 2 Clinical hospital "Pheophania", Kyiv, Ukraine

## Scientific objectives

In recent decades, *foresight* is widely used in developed countries for solving the problems of short-term and long-term planning and making the strategic decisions concerning industrial and economic development at the national, regional level and at the level of large organizations and companies [1]. One of the ten recognized at the global level threats is a threat to the quality of life and health of a person, it is expedient to build a strategy for the future of health care on the basis of the methodology of foresight. Using this methodology makes it possible to identify and justify the challenges of modern medicine and build the alternative scenarios to solve the accumulated problems in the field of the health care. The experience of the leading countries shows that the success of improving the quality of human life and health in modern conditions of globalization of the world economy is greatly facilitated by a high rate of innovation development of medical science and technology, a high level of competitiveness of the national high-tech products for the global healthcare market. To develop a long-term (up to 2020) innovative vision for health development the methodology of foresight is proposed to use [1].

## Technological approaches

Foresight is the basis for establishing and strengthening of such relations, promoting coordination and implementation of national and regional innovation systems, increasing their effectiveness. Foresight provides a mechanism to achieve *such a goal*. It promotes communication between members of the health care system, discussion issues with a long-term mutual interest, co-ordination of relevant policies and, in some cases - cooperation. In the process of solving the problems of foresight during the processing, analysis and structuring the initial information the approaches of artificial intelligence were proposed for the first time and used to automatically generation the survey forms and conduction the expert assessment procedure in on-line mode [2]. The software, which is the basis of this platform, is a distributed information system of decision-making process for the building of scenarios of the future, which combines a powerful mathematical tool and created on the basis of modern technologies flexible web-based user interface.

## Results interpretation

Here we consider the implementation of methodologies of foresight for PPPM, applicable in *Predictive* medicine, based on gathering significant amount of medical biomarkers, properly calculated by software and evaluated by experts in conditions of the absence of processed data. *Personalized* approach supposes optimization diagnostic and treatment strategies compound of subjective uncertain decision [3], made in lack of input parameters; the analysis for the procedure of risk’s prediction by selection of negative variants for timely forecast of interventional mistake [4]. *Preventive* approach includes planning of innovations in health care, remodeling the healthcare structure, management of uncertain decisions in health care. On its basis the systematic process of “identification” of future key technologies (critical technologies) is fulfilled to help the representatives of the superior governing bodies of state health services, private institutions and companies in establishing the most effective science policy and planning of its development.

## Outlook and expert recommendations

We recommend to implement the methodologies of foresight for enhancing national and regional systems of innovation development, it becomes crucial for innovative methodologies of modern society at the national or regional level and at the level of individual branches of medicine or large organizations and companies.
